# Seasonal variability of the fatty acid composition in *Aurelia aurita* (Cnidaria: Scyphozoa): implications for gelativore food web studies

**DOI:** 10.1093/plankt/fbaa026

**Published:** 2020-06-13

**Authors:** Vanessa Stenvers, Xupeng Chi, Jamileh Javidpour

**Affiliations:** 1 Faculty of Science and Engineering, University of Groningen, Nijenborgh 7, 9747 AG Groningen, The Netherlands; 2 Key Laboratory of Marine Ecology & Environmental Sciences, Institute of Oceanology, Chinese Academy of Sciences (IOCAS), Nanhai Road 7, Qingdao 266071, China; 3 Department of Biology, University of Southern Denmark, Campusvej 55, 5230-Odense, Denmark

**Keywords:** gelatinous zooplankton, gelativore, fatty acid, Baltic Sea

## Abstract

Jellyfish population play an important role in aquatic food chains, and many animals predate on this ‘mostly water containing’ organisms. However, what gelativores predators could gain from their prey is still poorly understood. This study provides insight into the nutritional value of the moon jelly (*Aurelia aurita*) by means of its fatty acid (FA) composition, while investigating seasonal variability and differences between its free-swimming life stages. A biweekly sampling was carried out in a temperate coastal ecosystem, the Kiel Fjord, Germany and during two consecutive years. FA profile of *A. aurita* showed significant seasonal variability, while mature medusae (due to reproductive tissues) possessed highest FA content. In addition, moon jelly contained several essential FAs (i.e. arachidonic acid, 20:4ω6; eicosapentaenoic acid, 20:5ω3; docosahexaenoic acid, 22:6ω3), which likely support predator’s vital physiological functions. Even though total FA contents proved to be low (7 × 10^−3^–34 × 10^−3^% per g dry weight), evidence supporting *A. aurita*’s capability to meet the dietary requirements of predators such as fish and crustaceans is provided. Finally, implications for gelativore and future food web configurations are discussed, while proposing that jellyfish are likely to be, and become, more than an opportunistic prey to many organisms.

## INTRODUCTION

Whether or not jellyfish populations are increasing globally due to human-induced environmental changes has been a recurrent topic of debate over the past decades (Richardson *et al*., 2009; Condon *et al*., 2012). Although some researchers suggest dense aggregations have become a symptom of perturbed ocean ecosystems (e.g. Jackson *et al*., 2001; Richardson *et al*., 2009), others attribute an ostensible increase to the lack of historical baseline data and/or consider them part of natural oscillations (e.g. Condon *et al*., 2012; 2013). Regardless of the line of evidence, jellyfish blooms tend to maintain a negative reputation, fuelled by an array of complaints ranging from interfering with recreationists in coastal areas to outcompeting commercially important fish stocks (Purcell *et al*., 2007; Richardson *et al*., 2009). When looking beyond this human-centered perspective, jellyfish actually play an important role within marine ecosystems and we are only beginning to understand how complex their role within aquatic food webs is (Fleming *et al*., 2015; Choy *et al*., 2017). In recent years, not only the effect of jellyfish on lower trophic levels has been reconsidered (Purcell, 2003; Fleming *et al*., 2015), but also their previously proposed role as trophic dead ends. Many animals (e.g. a deep sea cephalopod, marine birds and various fish species), for instance, have been shown to predate on live gelatinous zooplankton (Hoving and Haddock, 2017; Thiebot *et al*., 2017; Hays *et al*., 2018), while their dead remains are thought to form an important organic carbon input into deep sea ecosystems (Sweetman *et al*., 2014; Lebrato *et al*., 2019).

In spite of this important role, it is not completely understood what predators gain from their gelatinous prey, as jellyfish are commonly reported with only low caloric values, which would deem their role as a food source of minor importance (Arai, 2005; Doyle *et al*., 2007; Milisenda *et al*., 2014). Biomolecules that could provide insight into this gelativore feeding strategy are fatty acids (FAs), which have conventionally been used as trophic markers to elucidate the composition of jellyfish diets (Fukuda and Naganuma, 2001; Ying *et al*., 2012; Vansteenbrugge *et al*., 2016; Milisenda *et al*., 2018). FAs are vital components of cell membranes and play a crucial role in processes such as metabolism, signaling pathways, somatic growth and reproduction (Guschina and Harwood, 2009; Parrish, 2009). The three polyunsaturated FAs (i.e. PUFAs) that are generally considered most important for the latter functions are arachidonic acid (ARA, 20:4ω6), eicosapentaenoic acid (EPA; 20:5ω3) and docosahexaenoic acid (DHA; 22:6ω3), better known as omega (ω) 6 and 3 FAs (Parrish, 2009). Most animals, however, cannot biosynthesize these FAs and, therefore, have to rely on them through their dietary intake (Iverson, 2009). As a result, certain FAs convey information on the nutritional value and food quality of prey organisms. Nonetheless, FAs have rarely been considered within an ecological context to explain the dietary value of jellyfish.

A common jellyfish species known to frequently form mass aggregations is *Aurelia aurita* (Linnaeus, 1758). As with most jellyfish, bloom of this scyphomedusa are frequently seen as problematic as they can interfere with commercial fisheries or obstruct coastal cooling water inlets (Fukuda and Naganuma, 2001; Lucas, 2001). Nevertheless, considering that the ‘top predator role’ of jellyfish has recently been revised, new conclusions are also slowly drawn for this species. Ates (2017) for instance described several cases of predation by sea anemones, while even a fungiid coral has been noted to be an occasional feeder of live *A. aurita* (Alamaru *et al*., 2009). Population outbreaks could therefore become an important energy source for aquatic organisms, especially in a globally changing environment where an increasing abundance of jellyfish is thought to replace other prey items. Consequently, knowledge on the suitability of *A. aurita* as a food source*,* and jellyfish in general, is vital to understand how future outbreaks may promote gelativore feeding strategies as an adaptive behavior to anthropogenic food web perturbations. In this study, the nutritional value of *A. aurita* was investigated by means of its FA composition in two consecutive years. Furthermore, differences between *A. aurita*’s free-swimming life stages (i.e. ephyrae, immature and sexually mature medusae) were assessed.

## METHOD

### Sampling method

Samples of *A. aurita* were collected biweekly at a fixed sampling station in the Kiel Fjord (54°19.6986′N, 10°9.0304′E), Germany, from March 2015 till November 2016. This was carried out by vertically towing a WP3 net (enclosing a 1 m^2^ area, with a mesh size of 1 mm), from 18 to 0 meter depth. Hereafter, all jellyfish from the net were counted and weighed, their bell diameter was measured, and life stage was determined. Specimens could be classified as either ephyrae, immature or mature medusae, with the latter identified by the presence of gonadal tissue. When present, gonads were separated from the bell and oral arm tissues and analysed independently. Moreover, stomachs were removed to reduce bias caused by FAs, originating from ingested prey. Finally, samples were rinsed with either artificial seawater or filtered seawater (through a 64 μm sieve), weighed and stored in glass containers at −80°C until further processing. Furthermore, when *A. aurita* abundance was low, additional specimens were collected by hand net to either produce enough replicates for a given day or simply obtain samples when no specimens were caught with the WP3 net. These samples, however, were not included in the final abundance count. Finally, water temperature and salinity were measured for each sampling event using a CTD sensor (CTD 48M, Sea & Sun Technology GmbH, Trappenkamp, Germany).

### Fatty acid analysis

Before the FA analysis, all *A. aurita* samples were freeze-dried (Christ LOC-1m, Alpha 1-4, at 0.100 mbar and −50°C) for a minimum duration of 12 h, after which their total dry weight (DW) was determined. Because the biomass of the ephyrae was considerably low, specimens collected on a given day had to be pooled in order to obtain enough biomass. Moreover, since the samples in this study were part of a larger time series project, it was decided to only select three ephyrae sampling events for FA analysis, leaving the rest for stable isotopes analysis as part of a different manuscript. Subsequently, the samples were grinded into a powder and 2–7 mg of each sample was weighed for the analysis. If not immediately analysed, samples were stored at −80°C and freeze-dried again for 3 h to ensure no water accumulated before measuring their final DW. Before the FA composition could be determined, FAs had to be extracted and chemically altered first, which was carried out by following the method described by Chi *et al*. (2018) using the creation of fatty acid methyl esters (FAMEs) through acid transesterification. The FAMEs were then measured using a gas chromatograph (Trace GC-Ultra; Thermo Fisher Scientific Inc.) with hydrogen as its mobile phase, while being connected to a TR-FAME-column (10 m, with a diameter of 0.1 mm and 0.20 μm film) and a flame ionization detector. Subsequently, the gas chromatograph temperature program described by Arndt and Sommer (2014) was implemented. Lastly, the FAs were quantified using Chromcard software (Thermo Fisher Scientific Inc.) and peaks were identified on basis of their retention times together with the Supelco 37 FAME mixture. In addition to calculating the average total FA content in mg per g DW for each life stage, the average amount of saturated FAs (SFAs, i.e. no double bonds), monounsaturated FAs (MUFA, i.e. one double bond) and PUFA (i.e. ≥2 double bond) were calculated from the data. The obtained FA profiles were then linked to nutritional value by means of a literature review.

### Statistical analysis

Because immature medusae were almost continuously present during the sampling period, they provided the most representative basis and adequate sample size to investigate the seasonal variability of *A. aurita*’s FA composition. To assess this variability, a PERMANCOVA was used on basis of calculated Bray Curtis dissimilarities coefficients, pooling data for both 2015 and 2016 to focus on seasonal trends and as yearly differences were non-significant (PERMANCOVA, df = 1, pseudo F = 1.80, *R*^2^ = 0.029, *P* = 0.144). The covariates temperature and salinity were included, as it is well known that both these factors can influence FA composition in phytoplankton and zooplankton (Lichti *et al*., 2017), which are known prey sources of *A. aurita* (Javidpour *et al*., 2016). One of the assumptions for this test is an equal dispersion of data points (Anderson, 2001), which was tested using non-metric multidimensional scaling as the ordination method and a distance-based dispersion test (i.e. Levene’s test). Moreover, backwards elimination was applied to create the final and most parsimonious PERMANCOVA model, initially starting with all factors, covariates and interactions (i.e. seasons, temperature, salinity), after which non-significant terms (*P* > 0.05) were deleted. As a result, the final model encompassed the factor season and covariate salinity, together with their interaction. After this, the FAs that contributed most to the observed dissimilarities between seasons were calculated using the similarity percentages procedure similarity percentage analysis (SIMPER), which based its calculations on the Bray Curtis dissimilarities coefficients.

In order to assess differences between the free-swimming life stages, a PERMANOVA with Bray Curtis dissimilarity coefficients was implemented on the various tissue types (i.e. gonads, ephyrae, immature and mature medusae, pooling data from 2015 and 2016), after which the top contributing variables were again determined with SIMPER. Additionally, to visualize these differences, a principal component analysis (PCA) was used on the log (X + 1) transformed data, as the profiles deviated from normality. Lastly, the dietary ratios of ARA to EPA and DHA to EPA were calculated for each life stage. These ratios are important, as they can determine the mode of action and function of the latter FAs in marine animals, either promoting or limiting cellular and body function (Arts and Kohler, 2009; Zuo *et al*., 2012). Differences in group means were investigated with the non-parametric Kruskal–Wallis rank sum test (for each ratio separately) and post hoc Dunn’s test, as ratios showed some deviations from normality. All statistical procedures were conducted in the software R version 3.4.2 (R Core Team, 2017).

## RESULTS

### Abundance of *A. aurita*, temperature and salinity in the Kiel Fjord

From the beginning of the sampling period, *A. aurita* was present almost all year round, with a short absence during the winter season. In total, 89 ephyrae (of which 27 from 3 days were analysed for FAs), 62 immature medusae and 10 mature medusae were caught between 18 March 2015 and 1 November 2016. Moreover, one gonad sample was lost during sample preparation, resulting in a sample size of 9 for the reproductive tissue instead of the 10 as they were separated from the mature medusae. Immature medusae were generally found throughout the whole sampling period, while ephyrae predominated in late winter and mature medusa in summer (Supplementary Fig. 1). Moreover, the beginning of the year was marked by relatively high abundances, followed by a die-off or dispersion outside the Fjord in late summer. In 2015, the abundances of *A. aurita* reached peak densities in mid spring (i.e. day 91, 1.8 individuals/m^3^) with a second smaller peak at the end of spring (i.e. day 149, 0.8 individuals/m^3^). In 2016, peak abundances occurred in late winter (i.e. day 33, 2.2 individuals/m^3^), followed by a smaller bloom during mid-spring (i.e. day 103, 1.0 individuals/m^3^). Even though summer and fall were left with few individuals per cubic meter, the average size for these seasons was the greatest and individuals measured 11.2 ± 5.9 cm in bell diameter (i.e. compared with sizes of 0.5 ± 0.1 cm for winter and 2.4 ± 3.7 cm for spring). Furthermore, water temperature of the Kiel Fjord was highest in summer and fall (i.e. average 18.3 ± 2.4 and 14.5 ± 3.5°C, respectively) for both 2015 and 2016, with winter reaching average temperatures of 4.6 ± 1.8°C (Supplementary Fig. 2). Salinity showed more deviations between the years, with the lowest salinities measured from mid spring to mid fall for 2015 (ranging between 15 and 18), while salinities reached ~22 during approximately the same period in 2016 (Supplementary Fig. 2).

### Seasonal variability of FA composition

**Fig. 1:**
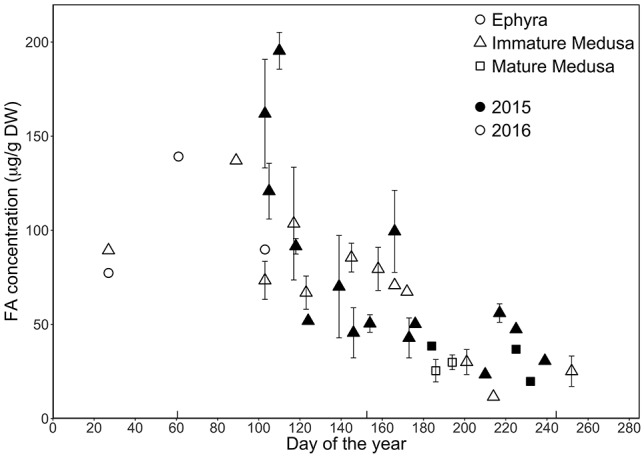
FA concentration in the bell and oral arm tissues of *A. aurita*, throughout 2015 and 2016 (given in μg/g DW per day of the year) (*n* = 75). The various life stages are indicated as follows: ephyrae are marked with circles, immature medusae by triangles and mature medusae by squares. The solid line represents 2015, while the dotted line indicates 2016. Seasons are indicated by the inverse ticks, with spring starting after day 60, summer after 152, fall after 244 and winter after 335.

Because the bell and oral arm tissue of medusae was analysed separately from the gonads, mature medusae in the following sections will only refer to medusae without their reproductive tissue. When comparing the total FA content in *A. aurita* throughout the year (i.e. gonads excluded)*,* concentrations proved to be the highest in winter and spring (i.e. coinciding with *A. aurita* peak abundances) and the lowest in summer and fall (Fig. 1). This pattern is roughly reflected by the seasonal occurrence of the different life stages, with ephyrae (102.08 ± 32.72 μg/g DW) and immature medusae (76.90 ± 45.05 μg/g DW) having higher FA contents than mature medusae (27.45 ± 8.13 μg/g DW; Table I). When calculating the percentage of FAs per unit DW, all life stages possessed only low values ranging between 0.001 and 0.021%. For ephyrae this resulted in an average FA percentage of 0.010 ± 0.003%, while immature medusae contained 0.008 ± 0.005% and mature medusae 0.003 ± 0.001%. In total, 19 FA could be identified.

**Table I:** Total average FA content of *A. aurita* and FA content of *Acartia tonsa*, *Mysis mixta* (in μg per g DW ± standard deviation) with average percentages of SFAs, MUFAs and PUFAs

Species, life stage/tissue type	Total μg per g DW	% SFA	% MUFA	% PUFA	Reference
*A. aurita,* ephyrae (*n* = 3)	102.08 ± 32.72	37.5	12.9	49.6	This study
*A. aurita,* immature medusae (*n* = 62)	76.90 ± 45.05	45.3	9.6	45.1	This study
*A. aurita,* mature medusae (*n* = 10)	27.45 ± 8.13	48.1	9.1	42.9	This study
*A. aurita,* gonads (*n* = 9)	341.42 ± 27.27	32.6	12.8	54.7	This study
*A. tonsa,* copepod	871.9 × 10^3^	40.3	18.5	41.3	Vanacor-Barroso *et al.* (2017)
*M. mixta,* female mysid shrimp	351.0 × 10^3^	22.3	39.4	36.4	Richoux *et al.* (2005)
*M. mixta,* male mysid shrimp	47.8 × 10^3^	19.0	26.1	53.1	Richoux *et al.* (2005)
*Sprattus sprattus,* sprat	88.25 × 10^3^^a^	31.3	39.6	29.1	Keinänen *et al.* (2017)
*Clupea harengus,* herring	50.60 × 10^3^^a^	33.0	33.7	33.2	Keinänen *et al.* (2017)

^a^Values are in μg per g wet weight.

**Fig. 2:**
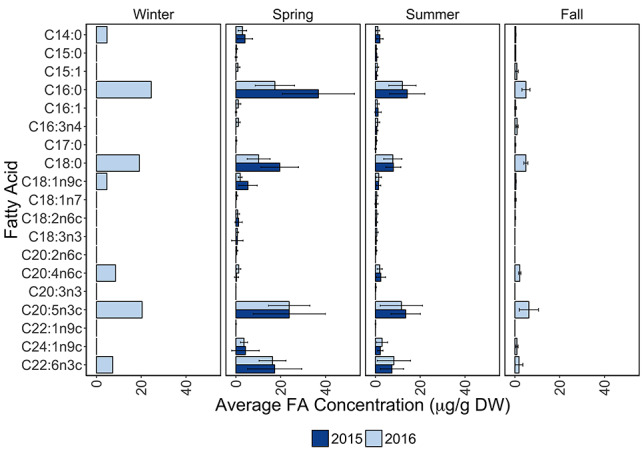
Overview FA profiles from immature *A. aurita*, grouped by season over the course of 2015 (dark gray) and 2016 (light gray) (given as the average concentration in μg/g DW) (*n* = 62). Error bars represent the range of each mean ± the standard error. Because winter only contained one immature medusa, standard errors could not be calculated for this season.

As already mentioned, immature medusae were present almost all year round and therefore provided the most appropriate sample size for assessing temporal differences. The final PERMANCOVA model tested season, salinity and their interaction, while temperature was excluded by backwards deletion. This was as expected, because meteorological seasons are distinguished by climate and therefore comprise the effect of temperature. Here, FA profiles of immature medusae differed significantly with season (PERMANCOVA, df = 3, pseudo F = 7.24, *R*^2^ = 0.253, *P* = 0.001). Salinity had a significant effect on the FA composition (PERMANCOVA, df = 1, pseudo F = 5.28, *R*^2^ = 0.062, *P* = 0.006), but could only account for 6% of the total variance. Furthermore, the effect of season did not depend on the effect of salinity, as the interaction between season and salinity was non-significant (PERMANCOVA df = 1, pseudo F = 2.85, *R*^2^ = 0.033, *P* = 0.056). SIMPER revealed that the dissimilarities between seasons were mainly caused by differences in palmitic acid (C16:0), stearic acid (C18:0), ARA (20:4ω6), EPA (C20:5ω3) and DHA (C22:6ω3) (Fig. 2, Supplementary Table 1).

### Ontogenetic variability of FA composition

Next to the seasonal trend, tissues of the various life stages also differed significantly from each other, including the separated gonads (PERMANOVA, df = 3, pseudo F = 17.60, *R*^2^ = 0.397, *P* = 0.001). The reproductive tissue clearly possessed highest FA concentrations (341.42 ± 27.27 μg/g DW), followed by ephyrae and immature medusae, with lowest quantities for the bell and oral arm tissues of mature medusae (Table I). SIMPER showed that at least 70% of these differences could be attributed to C16:0, C18:0, EPA and DHA (Table II). Additionally, the reproductive tissue possessed six FAs that were not present in the other tissues (i.e. C16:2ω4, C18:1ω9t, C20:0, C21:1ω9c, C22:0 and C24:0), although only occurring in minor amounts. The percentage of FA per unit DW for the gonads ranged between 0.03 and 0.04%, averaging at 0.03 ± 0.003%. When comparing the average amount of SFA, MUFA and PUFA present in the various life stages (Table I), all tissue types appeared to be characterized by relatively high levels of SFAs and PUFAs with lowest levels of MUFAs.

**Fig. 3:**
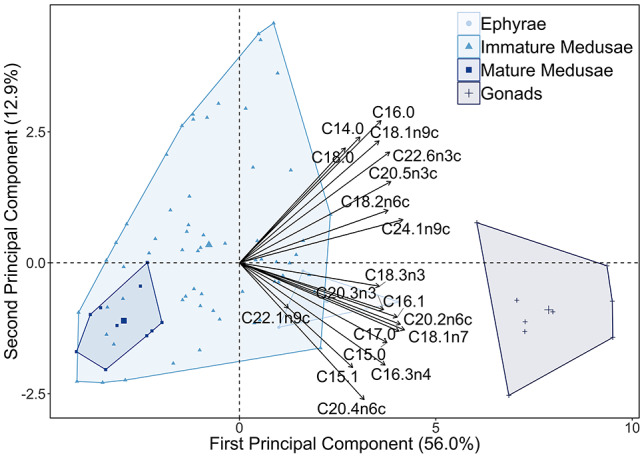
Principal component analysis for FA profiles of *A. aurita* (*n* = 84). Profiles are grouped according to tissue type of the life stages. The ephyrae (circles), immature (triangles) and mature medusa (squares) represent bell and oral arm tissues, while the gonads (plus sign) represent reproductive tissues separated from mature medusa. The slightly larger icons in the plot represent group means for each tissue type, while vectors indicate the various FA’s. The first principal component explains 56.0% of the variation, while the second principal component explains 12.9%.

The results produced by the PCA further substantiate the above observed patterns (Fig. 3). FA profiles of immature medusae showed a widespread distribution within the PCA plot, corresponding to the reported significant seasonal differences. Since immature medusae in winter and spring had relatively higher FA content than those in summer and fall (Fig. 2), part of the latter profiles was located in the FA-vector marked part the plot (i.e. indicating higher FA content). Ephyrae profiles also fell within this right half of the plot, while mature medusae had no such associations. Furthermore, the PCA confirmed that gonadal tissues were clearly distinct from all other tissues, as these profiles took a more secluded position in the FA-vector marked part of the plot. In addition to this, the PCA showed that the SIMPER-defined top contributing FAs (i.e. palmitic acid, stearic acid, EPA and DHA) were positively correlated, together with myristic (C14:0), oleic (C18:1ω9c), linoleic (C18:2ω6c) and nervonic acid (C24:1ω9c). The other remaining FAs (i.e. C15:0, C15:1, C16:1, C16:3ω4, C17:0, C18:1ω7, C18:3ω3, C20:2ω6c, C20:3ω3, C20:4ω6c and C22:1ω9c) also appeared to be correlated in the bottom right part of the plot. Moreover, the two principal component axes accounted for 68.9% of the total variation observed between the FA profiles.

**Table II:** SIMPER of FA that contributed ~70% of the observed variance when testing differences between free-swimming life stages of *A. aurita* using a PERMANCOVA

Contrasts between life stages/tissue types	Fatty acids	Cumulative percentage
Immature versus mature medusae	C20:5n3c	23.50
	C16:0	46.52
	C22:6n3c	64.54
Immature medusae versus ephyrae	C20:5n3c	22.93
	C16:0	39.52
	C22:6n3c	51.96
	C18:0	62.27
Immature medusae versus gonadal tissue	C20:5n3c	31.55
	C16:0	47.57
	C22:6n3c	62.80
Mature medusae versus ephyrae	C20:5n3c	26.85
	C16:0	43.30
	C22:6n3c	56.44
	C18:0	66.56
Mature medusae versus own gonadal tissue	C20:5n3c	30.90
	C16:0	48.37
	C22:6n3c	64.32
Ephyrae versus gonadal tissue	C20:5n3c	31.09
	C16:0	48.32
	C22:6n3c	64.89

**Fig. 4:**
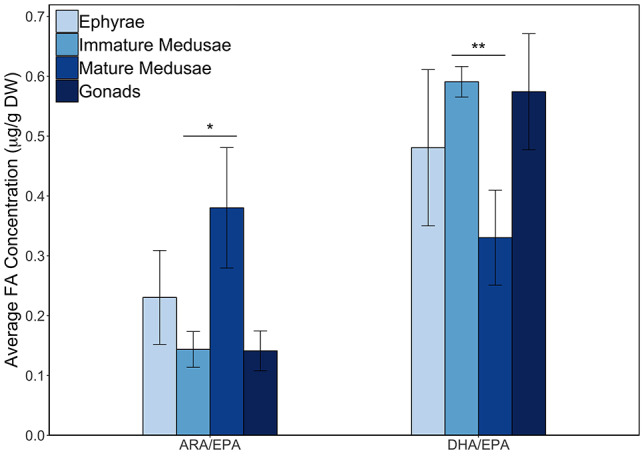
Dietary ratios for ARA/EPA and DHA/EPA in *A. aurita*, per life stage and tissue type, given as the average concentration in μg/g DW. Ephyrae are indicated by dim gray (*n* = 3), immature medusa by dark gray (*n* = 62), mature medusae by medium gray (*n* = 10) and gonads by light gray (*n* = 9). Error bars represent the range of each mean ± the standard error. Dissimilarities between life stages were caused by the bell and oral arm tissues of immature versus mature medusa, indicated by the asterisks (^*^Dunn’s test, *Z* = 3.287, *P* = 0.006 and ^*^^*^Dunn’s test, *Z* = 2.932, *P* = 0.020).

When calculating the dietary ratios ARA/EPA and DHA/EPA for all life stages, including the reproductive tissue, outcomes were always <1(Fig. 4). Average concentrations for ARA/EPA ranged from 0.14 to 0.38, with highest values for mature medusae and lowest values for the gonadal tissues and immature medusae. DHA/EPA took slightly higher values than ARA/EPA and ratios ranged between 0.33 and 0.59, with mature medusae possessing lowest outcomes and immature medusae highest. Furthermore, both fractions proved to differ significantly with tissue type (ARA/EPA Kruskal–Wallis df = 3, chi-squared = 13.67, *P* = 0.003; DHA/EPA Kruskal–Wallis df = 3, chi-squared = 8.95, *P* = 0.030). The post hoc analysis revealed that these dissimilarities were caused by the bell and oral arm tissues of immature versus mature medusae (ARA/EPA Dunn’s test, *Z* = 3.29, *P* = 0.006; DHA/EPA Dunn’s test, *Z* = 2.93, *P* = 0.020).

## DISCUSSION

Predation on jellyfish has been reported more frequently over the past years, but how predators benefit from the generally low caloric values is still poorly understood. Even though FAs have conventionally been used as trophic biomarkers for jellyfish, they have rarely been used to elucidate nutritional value for gelativore predators. Here, we used FA to demonstrate that mature medusae of the cosmopolitan species *A. aurita* had highest FA contents due to the presence of their gonads, making them the most nutritious prey item out of all free-swimming life stages. Moreover, the essential omega 3 and 6 FAs (EPA, DHA and ARA) were present in *A. aurita* throughout the whole study period with significant seasonal variability in FA profiles of immature medusae. In the following sections all findings will be discussed, after which we explore how these FAs may contribute to our understanding of predation on moon jelly.

### Temporal and ontogenetic variability of the FA composition in *A. aurita*

The observed seasonal differences are likely to be the result of varying prey abundances throughout the year (Fukuda and Naganuma, 2001; Fleming *et al*., 2015; Javidpour *et al*., 2016). Dietary shifts linked to differences in FA composition of *A. aurita* have previously been noted by Fukuda and Naganuma (2001) in the Seto Inland Sea, Japan, who showed that moon jelly will shift from a diatom to a detritus-based diet in late summer to fall. In the Kiel Fjord, similar changes have been reported by Javidpour *et al*. (2016), who used stable isotopes to show that *A. aurita* will move from a mesozooplankton to a seston-based diet during summer. This feeding pattern appears to be driven by the local plankton cycles, with a phytoplankton peak in early spring following the mesozooplankton bloom (Javidpour *et al*., 2009). Such a shift would fit the observed increase in total FA content of the moon jelly, which occurred approximately during the same period (Fig. 1). Nevertheless, it should be mentioned that *de novo* synthesis of FAs in jellyfish has not been investigated and FAs profiles might not be as dependent on prey as we think. This line of thinking is supported by Nichols *et al*. (2003), who revealed the unusual tetracosahexaenoic acid (THA, C24:6n3) in *A. aurita* from Western Australia, while hypothesizing that this FA might have been derived from internal DHA (C22:6n3) elongation. Consequently, there is reason to believe *A. aurita* is capable of *de novo* synthesis, although more research is needed to substantiate this.

When comparing the different life stages, it became evident that gonads were able to accumulate most FAs, while the somatic tissue appeared to have less lipid storage capacity. Indeed, most FAs in *A. aurita* are thought to be stored within cell membranes (Fukuda and Naganuma, 2001), which would limit overall lipid content. Only when gonads were present, medusae were able to deposit FAs. The increased FA concentrations found in the gonads are further supported by energetic measurements from Doyle *et al.* (2007), who measured caloric values in reproductive tissues of the medusozoa *Cyanea capillata* Linnaeus 1758*, Rhizostoma octopus* Gmelin 1791 and *Chrysaora hysoscella* Linnaeus 1767. In their investigations, authors found that gonads in the latter jellyfish will carry ~5 times more energy as the corresponding bell tissue. With this increased energetic value and FA content, it comes as no surprise that some animals will selectively prey on the gonadal tissue. Milisenda *et al*. (2014), for instance, showed that a fin fish, *Boops boops* Linnaeus 1758*,* will selectively prey on *Pelagia noctiluca* Forsskål 1775 containing gonads, while Thiebot *et al.* (2016) noticed the same for Adélie penguins on various species of gelatinous zooplankton. Besides providing more energy, the latter study also noted that the distinctive coloring of most gonads strengthens the tissue selectivity. Nevertheless, it should be recognized that the reproductive tissues only develop during selective months of the year (e.g. in the Kiel Fjord present in July and August), either weakening the predator-jelly feeding association outside of these months (Milisenda *et al*., 2014) or forcing gelativore to prey on whatever tissue is available.

### Nutritional value *A. aurita*

In this study, it became evident that *A. aurita* possessed only low overall FA content. For a fish predator such as Atlantic Cod (*Gadus morhua* Linnaeus 1758) from the Baltic Sea, which is known to be an occasional gelativore (Powles, 1958), other prey items including copepods, mysid shrimp and smaller fishes (Powles, 1958; Pachur and Horbowy, 2013) should provide thousand fold more FAs per gram of tissue weight (Table I). It thus seems *A. aurita* might not offer much in terms of total FA content, yet when comparing relative proportions of SFAs, MUFAs and PUFAs, moon jelly generally offers the highest percentages of PUFAs (42.9–54.7%). In the section that follows, it will be argued that PUFAs fulfill most important dietary requirements of fish predators, yet the question that first needs answering is how *A. aurita* can meet these requirements when only containing such low total FA quantities?

First, even though the total FA contents are low, when ingestion rates are high, they can compensate for the low FA content. This is illustrated by Arai *et al*. (Arai *et al*. (2003), who investigated chum salmon (*Oncorhynchus keta* Walbaum 1792) feeding on shrimp and the ctenophore *Pleurobrachia bachei* Agassiz 1860 (which is estimated to contain only 20% of the caloric value of crustaceans). Remarkably, the salmon digested *P. bachei* >20 times faster than the shrimp. Thus, when enough of the ctenophore was present, low caloric values could be compensated for (Arai *et al*., 2003). Following from this, is an explanation well phrased by Müller-Navarra (2008): ‘... food quality cannot be determined independently from food quantity, i.e., a low food quantity of high quality can serve the same as a high food quantity of low quality’.

Secondly, since jellyfish are capable of forming mass aggregations, they provide excellent opportunities for predators to gather considerable biomass. Jellyfish are relatively slow-moving animals and therefore allow for an energetic trade-off. Essentially, gelativore predators do not have to invest a lot of energy in catching their prey and the energy saved could then again make up for the low energy values of the jellyfish (Hays *et al*., 2018). An animal that seems to have taken this trade-off to its full advantage is the deep-sea octopus *Haliphron atlanticus* Steenstrup 1861. This octopus is associated with gelatinous prey items and was reported by Hoving and Haddock (2017) to have ‘very low mass-specific metabolic rates, comparable to some medusae’. Moreover, this advantage in energy expenditure was already formulated by Arai (1988), who noted that predators of coelenterates can save considerable energy when hunting these gradual moving animals.

Lastly, although we understand a healthy diet cannot be solely explained by FA content, it should be noted that many gelativores are generalist feeders (Cardona *et al*., 2012; Marques *et al*., 2016; Diaz Briz *et al*., 2017) and that jellyfish may supplement their diet. Conversely, some species are highly specialized and gelatinous zooplankton apparently does fulfill their complete nutritional requirements. An example of this are phyllosomas of the slipper lobster, which have been shown to survive and grow when exclusively fed with *Chrysaora pacifica* Goette 1886 and *A. aurita*, respectively (Wakabayashi *et al*., 2016). Subsequently, authors of this study reported that both jellyfish contained additional macronutrients and minerals that were essential for the development of the crustaceans, allowing for gelativore specialization. Other more well-known examples of animals that can sustain their dietary requirements with gelatinous zooplankton include the ocean sunfish and leatherback turtle, of which the latter even has adapted its gastrointestinal system to digest large amounts of the jelly biomass (Arai, 2005; Doyle *et al*., 2007). Similarly, Diaz Briz *et al*. (2017) found six fish species belonging to the suborder of the Stromateoidei to prefer a strictly gelatinous diet, with gastrointestinal adaptations convergent to the leatherbacks digestive system (e.g. conical denticles on the pharyngeal wall to grind prey, enlarged stomachs and a long digestive tracks).

Low total FA content therefore does not have to limit a gelativore and we shall now focus on what the FAs offer in terms of health benefits. As mentioned earlier, *A. aurita* carried the highest proportions of PUFAs compared with other prey types, with the essential FAs ARA, EPA and DHA present throughout the year. For predators of moon jelly, including fish and crustaceans, ARA and EPA are important because they function as precursors of eicosanoids, which are paracrine molecules regulating allergic reactions, inflammatory responses, immunity and neurological and reproductive functions (Tocher, 2010; Hill *et al*., 2012). Although ARA-derived eicosanoids are bioactive and induce inflammation, EPA-derived ones are less active and oppose inflammatory responses. In marine fish, both ARA and EPA can inhibit each other’s conversion, making the final outcome largely dependent on the ARA/EPA ratio present in the body (Arts and Kohler, 2009). Nevertheless, immunological responses are complex and optimal ratios vary with species, thereby making it difficult to assess the most beneficial ratio for the predator in question. Here, the investigated *A. aurita* life stages possessed only low ARA/EPA ratios and will thus limit the conversion of ARA into eicosanoids i.e. resulting in an anti-inflammatory response in a gelativore exclusively feeding on moon jelly (Fig. 4).

In addition to eicosanoids, multiple other health effects of the three PUFAs have been described. DHA is an important component of the retina in fish and other vertebrates, where it contributes to the membranes of rod cells and synaptic junctions (Sargent *et al*., 1999). In fish, both ARA and DHA are largely found throughout the brain, making them crucial components of regular neurological functioning (Tocher, 2010). In accordance with this, Zuo *et al.* (2012) reported several studies where DHA and EPA deficiency led to a decreased growth rate and survival, while postulating the idea that this deficiency led to cortical impairments, which worsened the effect by leading to malfunctions in hunting behavior. Furthermore, the ratio of DHA to EPA has been investigated extensively within aquaculture, where higher ratios (i.e. 2:1 or higher) proved most favorable with respect to enhanced growth, disease resistance and survival (Parrish, 2009; Zuo *et al*., 2012). *Aurelia aurita* from our study, however, always contained more EPA than DHA and therefore did not reach the desired aquaculture ratios (Fig. 4). Apart from fish, the importance of ARA, EPA and DHA have also been reported for several crustaceans, improving growth, survival and molting (Bell and Sargent, 2003).

Next to essential FAs, *A. aurita* also contained several SFAs at relatively elevated levels, of which the most notable were palmitic and stearic acid. Even though most animals are thought to be able to synthesize these two, they are an important source for energy metabolism (Sargent *et al*., 1999). Not only can FAs be readily absorbed by cells due to their hydrophobicity, enabling them to quickly reach their target locations, they also yield over twice as much energy compared to proteins and carbohydrates (Hill *et al*., 2012). Although palmitic and stearic acid were present in elevated amounts, other FAs (that are not directly used for the various functions mentioned above) can also be used for generating energy (Tocher, 2003). Consequently, *A. aurita* provides vital FAs for its fish and crustacean predators, supporting a multitude of physiological functions.

We can therefore conclude that jellyfish not only meet a predator’s energetic demands, but also the presence of macronutrients as reported by Wakabayashi *et al.* (2016) and key FAs as shown here indicate an important nutritional value of *A. aurita*. As such, present findings complement a remark made by Hays *et al*. (2018) in a recent review on the importance of jellyfish in ocean food webs, who emphasized that we need to start thinking beyond energetic gain when investigating jellyfish as prey and move toward nutritive value as part of food quality.

### Limitations, future research and implications

Even though this study was able to provide insight into *A. aurita*’s nutritional value with the help of FAs, it is important to remember that these FAs are largely influenced by diet. Therefore, FA profiles of moon jelly are not only likely to vary with location, but also with year. Even though non-significant, such inter annual differences could be seen in our data, where FA profiles of immature medusae differed slightly between 2015 and 2016. Furthermore, this study made use of the Supelco 37 FAME mixture, which possessed a standard mix of commonly found FAs, but which did not allow us to find the unusual THA (Nichols *et al*., 2003). Reason to believe that *A. aurita* from the Kiel Fjord might also have possessed this FA is because there were always some unidentified peaks left, which were not included in the Supelco 37 mix. Future research might therefore be aimed at investigating if *A. aurita* is able biosynthesise FAs and to what extent it can do this.

As the transition time from ephyrae to immature medusae is relatively short in a temporal area like the Kiel Fjord (Möller, 1980) and given a biweekly sampling in this study, we were limited in the total number of collected ephyrae specimens. Additionally, due to low biomass of single ephyra, it was needed to pool several individuals together for FA analysis. Therefore, we recommend more intense sampling for any future studies, especially if the focus should be on early planktonic life stage of jellyfish.

Ocean ecosystems are facing difficult times when it comes to human-induced environmental changes, exposing marine organisms to stressors like eutrophication, pollution, ocean acidification, overfishing, invasive species and climate change (Richardson *et al*., 2009; Doney *et al*., 2011). Subsequently, humans have succeeded to perturbate many marine food webs, forcing animals to seek alternative survival strategies (Jackson *et al*., 2001; Doney *et al*., 2011). Within the last years, growing evidence has shed light on the importance of gelatinous zooplankton within food webs and organisms are more often shown to be Gelativore feeders (Diaz Briz *et al*., 2017), may it be occasionally or by rule. Sometimes, even the most unthinkable cases of predation are reported, like the recent observed mallard ducks feeding on the anthomedusae *Velella velella* (Linnaeus 1758) (Phillips *et al*., 2017). Thus, whether jellyfish populations are increasing globally, they appear to provide a constant supply of biomass within many (perturbed) ecosystems and, at least in the case of *A. aurita,* allow for easy access to valuable FAs (Gibbons and Richardson, 2013). This makes it likely for gelatinous zooplankton to continue to serve as an important food source for many more organisms.

## CONCLUSION

Jellyfish populations prove to be more than just a nuisance to marine ecosystems. Despite the generally low caloric values of jellyfish, *A. aurita* has been shown to hold nutritional value with regard to its FAs and is clearly capable of supporting nutritional needs of various gelativores. Consequently, jellyfish blooms could prove to be an important food source for many animals, especially in a changing environment where the abundances of usual prey items might be endangered.

## Supplementary Material

Supplementary_Figures_fbaa026
