# Supplementary material for: Seasonal variability of the fatty acid composition in Aurelia aurita (Cnidaria: Scyphozoa): implications for gelativore food web studies
Source: J Plankton Res. 2020 Jun 13;42(4):440–52. doi: 10.1093/plankt/fbaa026 (PMC7344386; doi:10.1093/plankt/fbaa026)
Supplement: Supplementary_Figures_fbaa026 [file supplementary_figures_fbaa026.pdf]

Seasonal Variability of the Fatty Acid Composition in *Aurelia aurita* (Cnidaria: Scyphozoa): Implications for Gelativore Food Web Studies

Vanessa Stenvers[1], Xupeng Chi[2] and Jamileh Javidpour[3*]

1 Faculty of Science and Engineering, University of Groningen, The Netherlands

2 Key Laboratory of Marine Ecology and Environmental Sciences, Institute of Oceanology, Chinese Academy of Sciences, China

3 Department of Biology, University of Southern Denmark, Denmark

Supplementary material

S. Figure 1. Abundance of *A. aurita* in the Kiel Fjord throughout 2015 and 2016 (given in individuals/m^3 per day of the year) (n=236). Each point represents one sampling event, with the dominant life stages indicated as follows: ephyrae are marked by circles, immature medusae by triangles and mature medusae by squares. The solid line represents 2015, while the dotted line indicates 2016. Seasons are indicated by the inverse ticks, with spring starting after day 60, summer after 152, fall after 244 and winter after 335.

S. Figure 2. Average water temperature (in°C, shown in grey) and salinity (in PSU, shown in black) in the Kiel Fjord over the course of 2015 (solid lines) and 2016 (dotted lines). Seasons are indicated by the inverse ticks, with spring starting after day 60, summer after 152, fall after 244 and winter after 335.

S. Table 1. Similarity Percentage Analysis (SIMPER) of fatty acid that contributed up to 70% of the observed variance when testing differences between seasons for the immature *A. aurita* medusae, using a PERMANCOVA.

Supplementary Figure 1

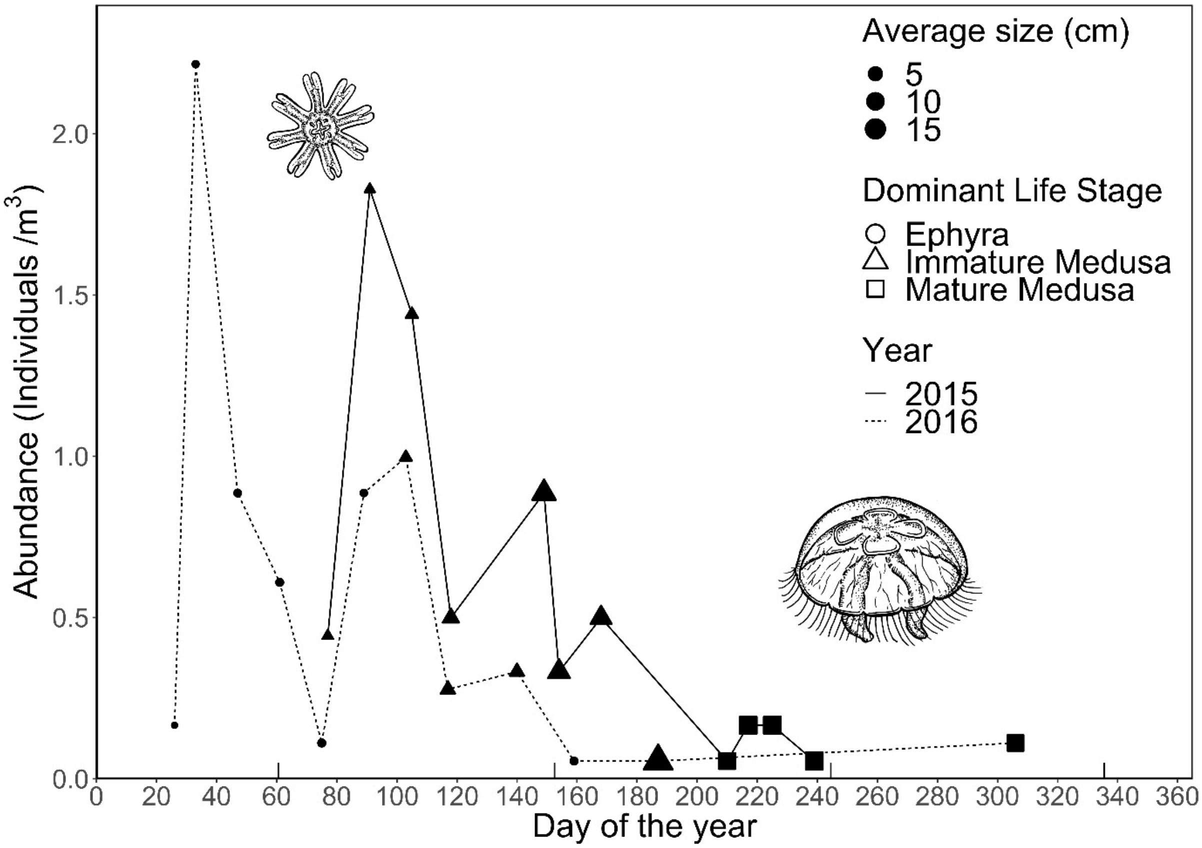

Supplementary Figure 2-

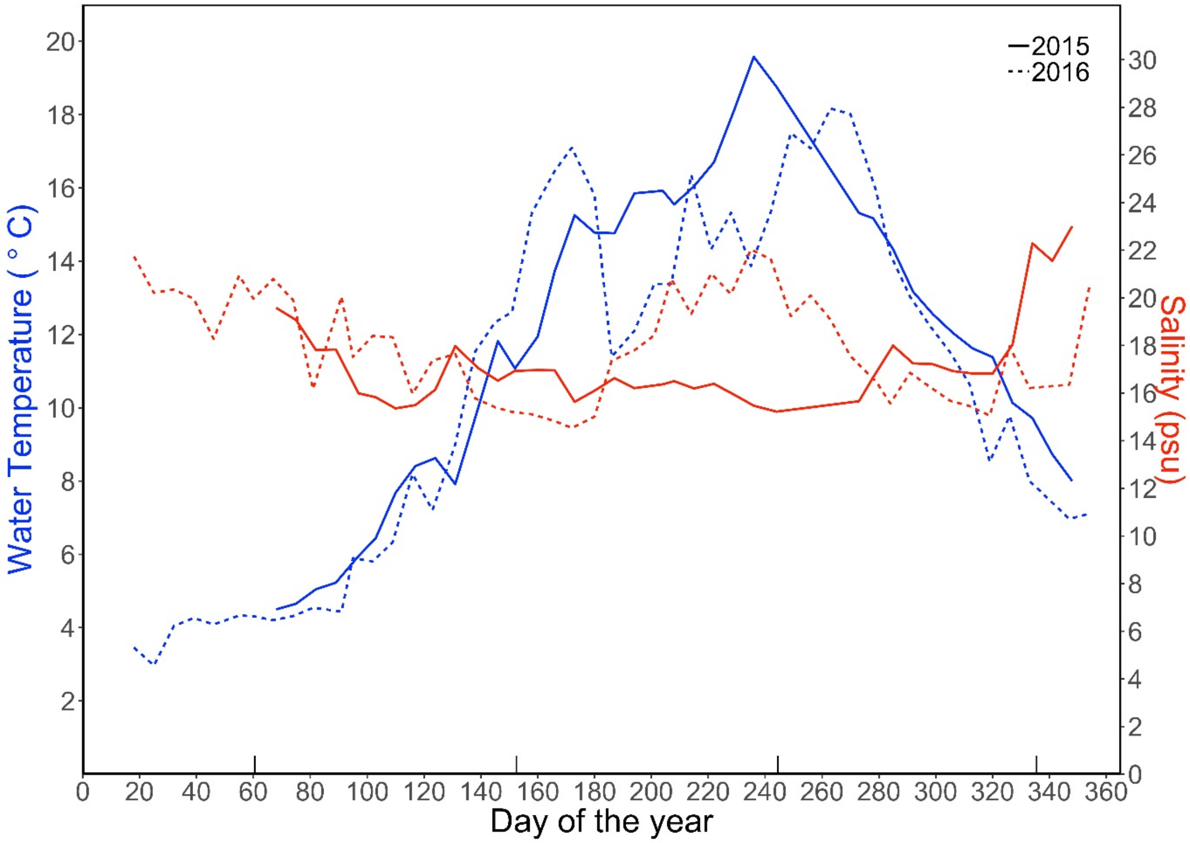

Supplementary Table 1

Contrasts between seasons	Fatty acids	Cumulative percentage
Spring versus Summer	C16:0	22.64
	C20:5n3c	45.09
	C22:6n3c	62.97
Spring versus Winter	C16:0	19.67
	C22:6n3c	35.05
	C20:5n3c	50.29
	C18:0	65.29
Spring versus Fall	C16:0	25.97
	C20:5n3c	48.30
	C22:6n3c	67.32
Summer versus Winter	C16:0	21.99
	C18:0	42.09
	C20:5n3c	58.81
	C22:4n6c	69.69
Summer versus Fall	C16:0	23.35
	C20:5n3c	45.88
	C22:6n3c	61.92
Winter versus Fall	C16:0	27.02
	C20:5n3c	46.79
	C18:0	66.51
